# The Relationship Between Calcaneal Bump Height and Progressive Collapsing Foot Deformity on Weight-Bearing Lateral Radiographs: Cross-Sectional Study in Adult Males

**DOI:** 10.3390/diagnostics16050745

**Published:** 2026-03-02

**Authors:** Hulya Cetin Tuncez, Selin Eroglu, Mahmut Tuncez, Zehra Hilal Adibelli

**Affiliations:** 1 Clinic of Radiology, Izmir City Hospital, Izmir 35540, Turkey; eroglu.selin2000@gmail.com (S.E.); adibellizehra@gmail.com (Z.H.A.); 2 Department of Orthopedics and Traumatology, Izmir Katip Celebi University Ataturk Training and Research Hospital, Izmir 35360, Turkey; drmahmuttuncez@gmail.com

**Keywords:** Progressive Collapsing Foot Deformity, adult flatfoot, calcaneus, Haglund deformity, bump height, hindfoot alignment

## Abstract

**Objectives:** To investigate the association between calcaneal bump height and hindfoot radiographic parameters on weight-bearing lateral radiographs in adult males with Progressive Collapsing Foot Deformity (PCFD), and to determine whether posterior calcaneal morphology differs between feet with and without PCFD-related flatfoot alignment. **Materials:** We retrospectively reviewed 583 men (1166 feet), aged 17–46 years, who underwent standing weight-bearing lateral foot radiographs between 1 January 2024 and 31 August 2025. Radiographic measurements included calcaneal pitch, Meary’s angle, navicular height, tibiocalcaneal angle, Böhler’s angle, Fowler–Philip angle, calcaneal bump height, and additional calcaneal morphological indices. A flatfoot alignment consistent with PCFD was defined as a calcaneal pitch < 18°. Receiver operating characteristic (ROC) analysis and multivariable logistic regression were performed to assess diagnostic performance and identify parameters independently associated with flatfoot alignment. **Results:** Flatfoot alignment was identified in 232 feet (19.9%) from 153 patients (26.2%). Compared with normally aligned feet, the flatfoot group demonstrated significantly lower navicular height, calcaneal bump height, and Böhler’s angle, along with higher tibiocalcaneal and Meary’s angles (all *p* < 0.001). ROC analysis showed navicular height to be the most accurate diagnostic parameter (AUC = 0.75), followed by the tibiocalcaneal angle (AUC = 0.69). Multivariable logistic regression revealed that navicular height ≤ 52.7 mm, tibiocalcaneal angle > 64.6°, Böhler’s angle ≤ 32.9°, Meary’s angle > 4.9°, calcaneal bump height ≤ 3.9 mm, and Fowler–Philip angle > 61.1° were independently associated with flatfoot alignment (Nagelkerke R^2^ = 0.293, *p* < 0.001). **Conclusions:** Calcaneal bump height is reduced in PCFD and reflects posterior calcaneal remodelling associated with hindfoot malalignment and medial arch collapse. Although not a primary diagnostic parameter, calcaneal bump height provides complementary morphological information that may inform surgical planning and osteotomy strategy aimed at restoring physiologic hindfoot biomechanics and Achilles tendon loading in patients with PCFD.

## 1. Introduction

Progressive Collapsing Foot Deformity (PCFD), previously referred to as flatfoot or pes planus, is a common condition observed in approximately 11–29% of males and females aged 18–25 years [1,2]. PCFD in adults describes a spectrum of deformities that persist or develop after skeletal maturity and are characterized by progressive collapse of the medial longitudinal arch, hindfoot valgus malalignment, and variable forefoot abduction. The clinical presentation of PCFD is heterogeneous, ranging from asymptomatic incidental findings to progressive, painful deformities associated with functional impairment. In adults, PCFD encompasses a broad range of pathological mechanisms, including persistence of congenital deformity, post-traumatic changes, degenerative processes, and conditions associated with systemic disease [3,4,5].

The calcaneus represents a critical structural component in the pathomechanics of PCFD. As the primary weight-bearing bone of the hindfoot, the calcaneus plays a fundamental role in supporting the medial longitudinal arch. Valgus deviation of the calcaneus at the subtalar joint, a hallmark feature of PCFD, alters hindfoot alignment and disrupts the normal biomechanics of the foot. In particular, calcaneal valgus shifts the moment arm of the tibialis posterior tendon, reducing its mechanical efficiency and contributing to progressive collapse of the medial arch and symptom development in adult PCFD [6].

Despite the central role of the calcaneus in PCFD, relatively little attention has been directed toward posterior calcaneal morphology in this condition. In contrast, Haglund deformity—characterized by retrocalcaneal bursitis and a bony prominence located at the posterosuperior aspect of the calcaneus—has been well described as a distinct pathological entity associated with posterior heel pain [7,8]. Whether calcaneal alignment changes seen in PCFD are associated with increased posterior calcaneal prominence or instead reflect a different pattern of calcaneal morphological adaptation remains poorly understood.

Understanding the relationship between calcaneal alignment and posterior calcaneal morphology is clinically relevant, as posterior heel pain and radiographic calcaneal changes are frequently encountered during the evaluation of patients with hindfoot deformity. Clarifying this relationship may help distinguish PCFD-related calcaneal adaptations from true Haglund deformity and prevent diagnostic misinterpretation.

Therefore, the purpose of this study was to investigate the relationship between calcaneal bump height and hindfoot alignment in adults with Progressive Collapsing Foot Deformity using weight-bearing lateral radiographs.

## 2. Materials and Methods

This retrospective study was approved by our university’s Institutional Review Board (IRB0379; 13 August 2025), and the records of patients who underwent standing, weight-bearing lateral foot radiographs between 1 January 2024, and 31 August 2025, were retrospectively reviewed. As no direct contact with participants occurred and all data were analyzed anonymously, the requirement for informed consent was waived by the ethics committee.

### 2.1. Patient Inclusion

**Figure 1:**
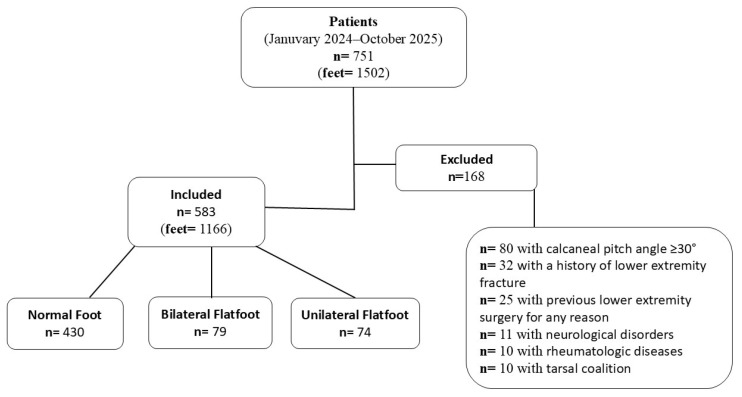
Patient flow chart.

A total of 751 individuals who underwent standing weight-bearing lateral foot radiography were initially screened for eligibility. Of these, 168 were excluded due to conditions that could affect radiographic measurements (Figure 1). Consequently, 583 patients, corresponding to 1166 feet, were included in the final analysis.

### 2.2. Evaluation of Patients

For all patients, demographic data were recorded, and standing weight-bearing radiographs of the feet were evaluated. On these radiographs, the following parameters were measured and documented: calcaneal pitch angle, calcaneal bump height, Meary’s angle, navicular height, Fowler–Philip angle, Böhler’s and Gissane angles, tibio-calcaneal angle, as well as calcaneal height and length.

In this study, the term “flatfoot” was used as a radiographic alignment descriptor to represent collapse of the medial longitudinal arch, rather than as a diagnostic label. In accordance with contemporary nomenclature, this alignment pattern is considered a radiographic manifestation consistent with PCFD. Specifically, reduced calcaneal pitch on weight-bearing lateral radiographs reflects medial arch collapse, which constitutes a core structural component of PCFD.

**Figure 2:**
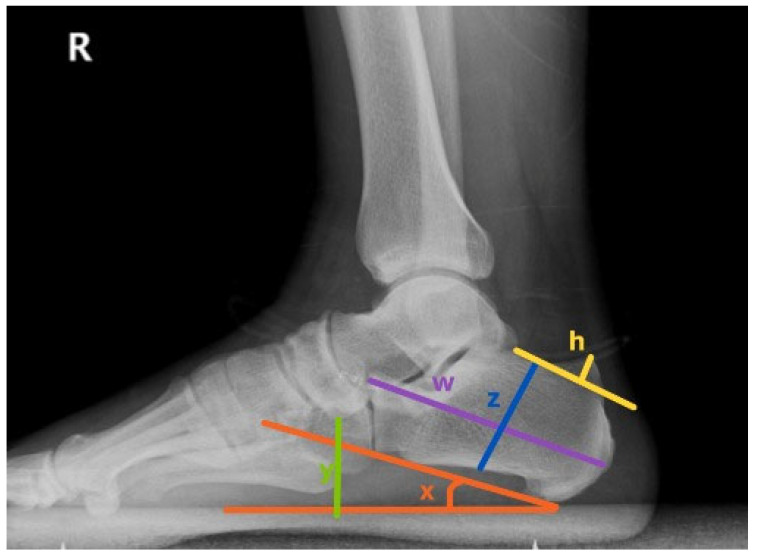
Radiographic measurement of foot. (h: Bump height, w: calcaneal length, x: calcaneal pitch angle, y: navicular height, z: calcaneal height).

The calcaneal pitch angle (CPA) represents the angle formed between the supporting surface and a line drawn tangential to the inferior aspect of the calcaneus (Figure 2). CPA was interpreted according to established radiographic criteria, with values below 18° considered consistent with flatfoot alignment on weight-bearing lateral radiographs, as previously described in imaging-based studies of pes planus and adult acquired flatfoot deformity [9,10].

**Figure 3:**
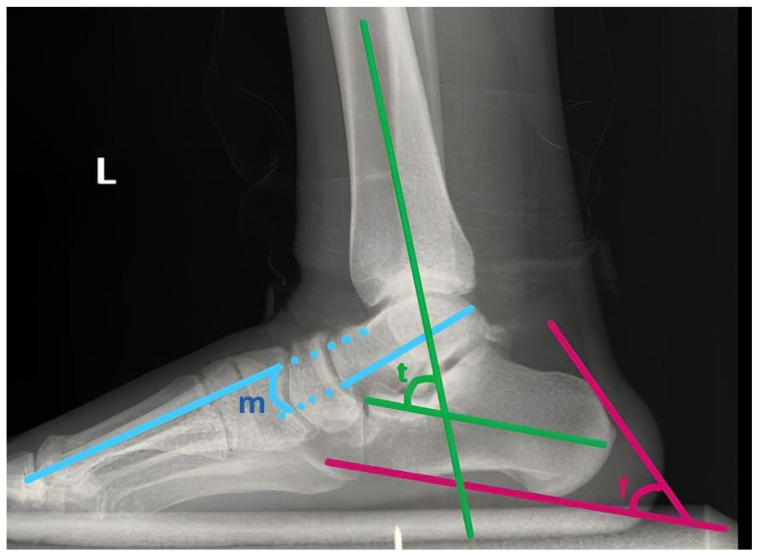
Radiographic measurement of foot. (f: Fowler and Flippe angle, m: Meary angle, t: Tibiocalcaneal angle).

The talo-first metatarsal angle, also referred to as Meary’s angle, is determined by the intersection between the longitudinal axis of the talus and that of the first metatarsal (Figure 3). In a neutral or rectus foot, these lines are parallel, resulting in an angle of approximately 0°. An angle greater than 4° with the apex directed inferiorly signifies a flatfoot alignment, while an angle greater than 4° with the apex oriented superiorly corresponds to a cavus foot.

The calcaneal bump height was measured as the vertical distance between a line tangential to the posterior calcaneal bump and the most prominent point of the posterosuperior calcaneal surface (Figure 2). This parameter reflects the degree of bony prominence associated with Haglund deformity. Increased bump height has been correlated with retrocalcaneal bursitis and insertional Achilles tendinopathy.

Navicular height was defined as the perpendicular distance between the lowest point of the navicular bone and the supporting surface on a weight-bearing lateral radiograph (Figure 2). It represents the height of the medial longitudinal arch. Decreased values indicate arch collapse, commonly observed in flatfoot alignment.

The Fowler–Philip angle was formed between a line along the superior surface of the calcaneal bump and another line drawn along the posterior surface of the calcaneus (Figure 3). The normal range was accepted as 44–69°. An angle exceeding 75° was considered indicative of a prominent posterosuperior calcaneus (Haglund deformity).

Böhler’s angle was measured between a line connecting the highest point of the anterior process and the posterior articular facet, and a second line connecting the posterior facet with the superior border of the calcaneal bump. The normal value was considered between 20° and 40°. A decrease below 20° suggested depression of the posterior facet or calcaneal fracture.

Gissane’s angle, also known as the critical angle of Gissane, was measured between the downward and upward slopes of the calcaneal superior surface at the anterior margin of the posterior facet. The normal range was accepted as 120–145°. Alteration of this angle was interpreted as an indicator of joint surface collapse or deformity.

The tibiocalcaneal angle was defined as the angle between the longitudinal axes of the tibia and the calcaneus on the lateral radiograph (Figure 3). It was used to assess hindfoot alignment. Increased or decreased values indicated varus or valgus deformity, respectively.

Calcaneal height was measured as the vertical distance from the most inferior point of the calcaneal bump to the highest point of the posterior articular surface. Calcaneal length was defined as the distance between the posterior bump and the anterior process. Both parameters were used to evaluate calcaneal morphology and structural alterations (Figure 2).

### 2.3. Statistics

In this study, IBM SPSS Statistics 26.0 was used for data analysis. Descriptive statistics calculated mean ± standard deviation, median (minimum–maximum), and interquartile range (IQR (Q1–Q3)) for continuous variables. Categorical variables are presented as frequency (n) and percentage (%).

Data conformity to a normal distribution was assessed using the Kolmogorov–Smirnov and Shapiro–Wilk tests, and skewness and kurtosis coefficients were examined. In comparisons of two independent groups, the Independent Samples *t*-test was used for normally distributed parameters, and the Mann–Whitney U test was used for non-normally distributed parameters. In comparisons of three or more independent groups, one-way analysis of variance (ANOVA) was used for normally distributed data, and the Kruskal–Wallis test was used for non-normally distributed data. Multiple comparisons after the ANOVA were performed with Bonferroni correction.

Research curve analysis was performed to assess diagnostic performance, and the most appropriate cutoff points were determined according to the Youden index. In interpreting AUC values, values of 0.90–1.00 were considered “excellent,” 0.80–0.89 “good,” 0.70–0.79 “moderate,” 0.60–0.69 “poor,” and 0.50–0.59 “unsuccessful.”

The chi-square test was used to evaluate the relationships between categorical variables, and odds ratios were calculated.

Multivariate logistic regression analysis was performed to identify risk factors for flat feet. Flat feet status (Yes/No) was used as the dependent variable, and radiographic parameters categorized according to the cutoff points determined in the ROC analysis were included as independent variables. The backward Wald method was used in model selection, and the significance threshold was set at *p* < 0.05. The model’s goodness of fit was assessed using the Hosmer–Lemeshow test, and model performance was reported using −2 Log likelihood, Cox & Snell R^2^, and Nagelkerke R^2^ values.

Pearson correlation analysis was performed to examine the relationships between variables. Correlation coefficients were interpreted as r < 0.30 as “very low,” 0.30–0.49 as “low,” 0.50–0.69 as “moderate,” 0.70–0.89 as “high,” and ≥0.90 as “very high.”

Multiple linear regression analysis was performed to determine predictors of calcaneal bump height, and backward elimination was used for model selection.

Multicollinearity in regression models was assessed using Variance Inflation Factor (VIF) and Tolerance values, with VIF < 5 and Tolerance > 0.2 being the criteria.

In all analyses, statistical significance was set at *p* < 0.05, and the confidence interval was set at 95%.

Radiographic measurements were performed at the foot level, and each foot was treated as a separate analytical unit because alignment parameters and calcaneal morphology were evaluated independently for each side. However, potential within-subject correlation between bilateral feet cannot be completely excluded and was considered during interpretation of the findings.

## 3. Results

A total of 583 male participants (1166 feet) were included in the study, with a mean age of 24 ± 4 years (range: 17–46 years). The study population consisted entirely of young-to-middle-aged adult males. Radiographic assessment revealed a mean calcaneal pitch angle of 21.3 ± 4.2°, mean navicular height of 53.7 ± 7.1 mm, and mean tibiocalcaneal angle of 61.5 ± 9.2°. Measurement values for the remaining foot parameters are presented in detail in Table 1.

Based on the calcaneal pitch angle criterion (<18°), flatfoot alignment was identified in 232 feet (19.9%). On an individual basis, flatfoot alignment was present in 153 men (26.2%), of whom 74 (12.7%) exhibited unilateral and 79 (13.6%) bilateral involvement (Figure 1).

Statistical analyses demonstrated significant alterations in most parameters among flatfooted feet. The most pronounced difference was observed in navicular height, which was on average 7 mm lower in the flatfoot alignment group compared with the normal group (*p* < 0.001) (Table 2). Similarly, calcaneal bump height was significantly reduced in the flatfoot alignment group (*p* < 0.001). Regarding angular parameters, the tibiocalcaneal angle was markedly greater in the flatfoot alignment group (66 ± 8°) compared with the normal group (60 ± 9°) (*p* < 0.001). The Meary’s angle was also higher in the flatfoot alignment group [median 4.1 (2.1–8.1)] than in normal feet [3.1 (1.8–5.1)] (*p* < 0.001). Conversely, calcaneal length (95 ± 7 mm vs. 94 ± 7 mm, *p* = 0.165) and Gissane’s angle (105 ± 9° vs. 104 ± 9°, *p* = 0.439) showed no statistically significant differences between groups (Table 2).

Receiver operating characteristic (ROC) curve analysis was performed to evaluate the diagnostic performance of radiographic parameters for the classification of flatfoot alignment. Navicular height demonstrated the highest diagnostic accuracy [AUC = 0.75, 95% CI: 0.724–0.774, *p* < 0.001] (Table 3). The optimal cutoff point for this parameter was determined as ≤52.7 mm, yielding 72% sensitivity and 65% specificity. The tibiocalcaneal angle showed moderate diagnostic performance with an AUC value of 0.694 [95% CI: 0.667–0.721, *p* < 0.001]. Among the remaining parameters, calcaneal bump height (AUC = 0.607), Meary’s angle (AUC = 0.591), Fowler and Philip angle (AUC = 0.592), Böhler’s angle (AUC = 0.588), and calcaneal height (AUC = 0.578) were statistically significant but demonstrated weak diagnostic performance. Conversely, calcaneal length (AUC = 0.53, *p* = 0.158) and Gissane’s angle (AUC = 0.526, *p* = 0.216) did not reach statistical significance (Table 3).

Multivariate logistic regression analysis using the backward Wald method identified six independent variables that remained significant in the final model (Table 4). The model was statistically significant [χ^2^(6) = 238.68, *p* < 0.001], explaining 29.3% of the variance in flatfoot occurrence (Nagelkerke R^2^ = 0.293). The goodness-of-fit test was non-significant [Hosmer–Lemeshow χ^2^(8) = 9.48, *p* = 0.303], indicating an adequate model fit. The strongest predictor of flatfoot alignment was navicular height ≤ 52.7 mm. Other significant predictors included tibiocalcaneal angle > 64.6°, Böhler’s angle ≤ 32.9°, Meary’s angle > 4.9°, calcaneal bump height ≤ 3.9 mm, and Fowler and Philip angle > 61.1° (Table 4). Calcaneal height was excluded from the model due to non-significance (*p* = 0.770).

Subgroup analysis comparing unilateral and bilateral flatfoot alignment cases revealed that both subgroups exhibited significantly lower calcaneal bump height, calcaneal height, navicular height, and Böhler’s angle than the non-flatfoot group (*p* < 0.001) (Table 5). Additionally, the flatfoot alignment group demonstrated significantly greater tibiocalcaneal, Fowler and Philip, and Meary’s angles (*p* < 0.001). Post hoc analyses indicated that these differences were most pronounced between the bilateral flatfoot alignment and non-flatfoot groups. Feet in the bilateral group showed significantly lower navicular height, greater tibiocalcaneal angle, and greater Meary’s angle compared with non-flatfooted feet. In contrast, unilateral flatfoot alignment exhibited significant differences in navicular height (51 ± 7 mm) and tibiocalcaneal angle (65 ± 8°) compared with non-flatfooted feet (*p* < 0.001), but not in Meary’s angle (*p* = 0.536). No statistically significant differences were observed among groups for calcaneal length and Gissane’s angle (Table 5).

## 4. Discussion

The present study investigated the relationship between calcaneal bump height and hindfoot radiographic parameters in young-to-middle-aged adult males with PCFD using weight-bearing lateral radiographs. Our findings demonstrated that feet exhibiting medial longitudinal arch collapse consistent with PCFD showed a significant reduction in calcaneal bump height, navicular height, and Böhler’s angle, accompanied by a marked increase in tibiocalcaneal and Meary’s angles, compared with feet with normal alignment. Among all evaluated radiographic variables, navicular height demonstrated the highest diagnostic accuracy for identifying PCFD-related arch collapse, whereas calcaneal bump height—despite being independently associated with PCFD—showed only modest diagnostic performance (AUC = 0.607). Collectively, these findings indicate that posterior calcaneal morphology is altered in PCFD and should be interpreted within the broader context of hindfoot malalignment and medial arch collapse.

The strong association between reduced navicular height and PCFD observed in this study is consistent with prior work identifying medial longitudinal arch collapse as a defining structural feature of the deformity [3,6]. Previous investigations by Richie [5] and Jones and Todd [4] similarly emphasized that decreased navicular height and increased Meary’s angle reliably reflect loss of medial arch integrity. Our novel observation of reduced calcaneal bump height in feet with PCFD contrasts with the traditional emphasis on increased posterior calcaneal prominence seen in conditions such as Haglund deformity (HD). While earlier studies of HD reported variable relationships between posterior calcaneal morphology and symptoms [7], more recent work by Tang et al. introduced calcaneal bump height and bump–calcaneus ratio as objective radiographic parameters, reinforcing the clinical relevance of calcaneal morphometrics [8]. In contrast to HD, our data suggest that PCFD is associated with posterior calcaneal flattening rather than prominence, reflecting a distinct morphological adaptation linked to hindfoot valgus and altered loading mechanics.

From a biomechanical perspective, valgus deviation of the calcaneus in adult PCFD may alter ground-reaction force vectors and reduce posterior–superior traction forces that have been associated with the development of a conventional Haglund-type prominence [11,12,13]. This biomechanical mechanism may partly explain the lower calcaneal bump height and reduced Fowler–Philip angles observed in the flatfoot alignment group in our cohort. Conversely, cavus alignment has been associated with increased Achilles tendon tension and posterior–superior loading, which may contribute to hypertrophic remodeling of the posterior calcaneus [14]. Together, these findings suggest a potential inverse morphological relationship between flatfoot alignment and Haglund-type posterior calcaneal prominence; however, the present radiographic study design does not allow causal inference.

Surgical correction strategies for PCFD, including medial displacement calcaneal osteotomy, lateral column lengthening, and combined procedures, primarily aim to restore hindfoot alignment and medial arch structure [14,15,16,17,18,19,20,21,22]. Although these procedures address global foot biomechanics, their relationship with posterior calcaneal morphology remains incompletely understood. The current findings should therefore be interpreted as providing morphological observations that may inform future biomechanical and clinical research rather than direct surgical recommendations. Further prospective studies integrating clinical outcomes and functional assessment are needed to determine whether variations in calcaneal bump height have any measurable relevance in surgical planning or postoperative biomechanics.

Consistent with existing literature, navicular height emerged as the strongest independent predictor of PCFD-related medial arch collapse in our cohort, with a threshold value of ≤52.7 mm demonstrating the highest diagnostic utility. Williams et al. validated normalized truncated navicular height as a reliable clinical indicator of flatfoot alignment, reporting excellent diagnostic accuracy in pediatric populations [23]. Similarly, Jung et al. demonstrated strong correlations between navicular height reduction and flexible flatfoot deformity using three-dimensional foot-scanning techniques [24]. Our findings extend this body of evidence by confirming the central role of navicular height in radiographic assessment of PCFD in young-to-middle-aged adults.

Subgroup analyses revealed that both unilateral and bilateral PCFD-related arch collapse were associated with significant reductions in calcaneal bump height, calcaneal height, navicular height, and Böhler’s angle, along with increased tibiocalcaneal, Fowler–Philip, and Meary’s angles. These changes reflect the complex, multiplanar nature of PCFD, encompassing medial column collapse and progressive hindfoot valgus. The more pronounced alterations observed in bilateral cases suggest a greater degree of structural adaptation and deformity severity. Although studies directly comparing unilateral and bilateral adult PCFD remain limited, existing literature indicates that bilateral involvement is common and may represent a more advanced stage along a progressive deformity spectrum, with unilateral cases potentially reflecting earlier or less severe disease [24,25,26]. Reviews of adult-acquired flatfoot further support this concept, emphasizing the progressive nature of PCFD and its tendency toward bilateral involvement over time [25,26,27,28].

This study has several strengths, including a large, homogeneous cohort, standardized weight-bearing radiographic assessment, and robust multivariable modeling to identify independent associations with PCFD-related alignment changes. Nevertheless, certain limitations must be acknowledged. The retrospective, cross-sectional design precludes assessment of temporal progression or causality. The exclusive inclusion of young-to-middle-aged adult males limits generalizability to female or older populations, in whom PCFD may demonstrate different clinical and morphological characteristics. Additionally, the lack of correlation with clinical symptoms—particularly Achilles or retrocalcaneal pain—prevents direct assessment of the functional implications of reduced calcaneal bump height. Furthermore, intra- and interobserver reliability analyses for radiographic measurements were not performed, which may introduce measurement variability despite the use of standardized radiographic techniques. As both feet from some participants were included and each foot was treated as an independent analytical unit, potential within-subject correlation may represent a source of statistical non-independence.

## 5. Conclusions

In conclusion, this study demonstrates that PCFD in young-to-middle-aged adult males is associated with decreased calcaneal bump height and reduced navicular height, reflecting both structural and morphological alterations of the hindfoot. Although the standalone diagnostic contribution of calcaneal bump height is limited when compared with navicular height, its inverse association with PCFD-related medial arch collapse suggests a biomechanical adaptation rather than a coexisting Haglund-type deformity. These findings indicate that posterior calcaneal morphology may represent an additional radiographic feature warranting further investigation in the context of PCFD. The observed calcaneal morphological changes further support the concept that chronic hindfoot valgus and medial arch collapse may result in progressive posterior calcaneal remodeling over time. While confirmation in larger, prospective, multicenter cohorts is warranted, the present findings may facilitate future research exploring the potential clinical and surgical relevance of posterior calcaneal morphology in PCFD.

## Figures and Tables

**Table 1:** Descriptive statistics of radiographic parameters (foot = 1166).

Parameter	Foot	Mean ± SD	Median (Min–Max)	IQR (Q1–Q3)
Calcaneal Bump Height	1166	3.9 ± 1.3	3.8 (0.8–8.1)	3.0–4.8
Calcaneal Height	1166	44.6 ± 3.5	44.5 (35.5–55.8)	42.1–47.2
Calcaneal Length	1166	94.3 ± 6.7	94.0 (77.5–116.4)	89.4–98.9
Navicular Height	1166	53.7 ± 7.1	54.0 (31.7–73.1)	49.5–58.5
Calcaneal Pitch Angle	1166	21.3 ± 4.2	21.5 (4.7–30.0)	18.7–24.4
Tibio-calcaneal Angle	1166	61.5 ± 9.2	61.4 (35.8–87.9)	55.7–67.4
Bohler’s Angle	1166	34.6 ± 5.6	34.6 (18.1–49.1)	30.7–38.6
Fowler and Philip Angle	1166	59.9 ± 6.2	59.9 (38.5–77.2)	56.1–64.2
Gissane’s Angle	1166	104.4 ± 8.7	104.6 (75.9–130.4)	98.5–110.4
Meary’s Angle	1166	4.5 ± 3.9	3.2 (0.1–21.3)	1.9–6.0

**Table 2:** Radiographic parameters according to flatfoot status-foot-based analysis. (* Independent Samples *t* Test).

Parameter	Total (Foot = 1166)	Flat Foot (Foot = 232)	Normal (Foot = 934)	*p* *
	Mean ± SD	Mean ± SD	Mean ± SD	
Calcaneal Bump Height	3.9 ± 1.3	3.5 ± 1.3	4.0 ± 1.2	<0.001
Calcaneal Height	45 ± 4	44 ± 3	45 ± 4	<0.001
Calcaneal Length	94 ± 7	95 ± 7	94 ± 7	0.165
Navicular Height	54 ± 7	48 ± 7	55 ± 6	<0.001
Tibio-calcaneal Angle	62 ± 9	66 ± 8	60 ± 9	<0.001
Bohler’s Angle	35 ± 6	33 ± 6	35 ± 6	<0.001
Fowler and Philip Angle	60 ± 6	62 ± 6	60 ± 6	<0.001
Gissane’s Angle	104 ± 9	105 ± 9	104 ± 9	0.439
Meary’s Angle *	3.2 (1.9–6.0)	4.1 (2.1–8.1)	3.1 (1.8–5.1)	<0.001 *

**Table 3:** Diagnostic performance of radiographic parameters for flatfoot classification (ROC curve analysis).

Parameter	AUC (95% CI)	*p*-Value	Youden Index	Cut-Off Value	Sensitivity (%)	Specificity (%)
Navicular Height	0.75 (0.724–0.774)	<0.001	0.377	≤52.7	72	65
Tibiocalcaneal Angle	0.694 (0.667–0.721)	<0.001	0.298	>64.6	59	71
Calcaneal Bump Height	0.607 (0.578–0.635)	<0.001	0.177	≤3.9	67	51
Meary’s Angle	0.591 (0.562–0.619)	<0.001	0.195	>4.9	45	74
Fowler and Philip Angle	0.592 (0.563–0.621)	<0.001	0.155	>61.1	54	61
Bohler’s Angle	0.588 (0.559–0.616)	<0.001	0.153	≤32.9	51	64
Calcaneal Height	0.578 (0.549–0.606)	<0.001	0.116	≤44.2	57	55
Calcaneal Length	0.53 (0.501–0.559)	0.158	0.064	>89.8	78	28
Gissane’s Angle	0.526 (0.497–0.555)	0.216	0.083	>105.4	52	56

**Table 4:** Multivariate logistic regression analysis for predictors of Flatfoot. (Method: backward stepwise (Wald). −2 Log likelihood: 924.92, Cox & Snell R^2^: 0.185, Nagelkerke R^2^: 0.293, Hosmer–Lemeshow Test: χ^2^(8) = 9.48, *p* = 0.303, overall classification accuracy: 82.8%).

Predictor	B	SE	Wald	*p*-Value	Odds Ratio (95% CI)
Navicular Height (≤52.7)	1.68	0.18	89.24	<0.001	5.35 (3.78–7.57)
Tibiocalcaneal Angle (>64.6)	1.14	0.17	47.00	<0.001	3.14 (2.26–4.35)
Bohler’s Angle (≤32.9)	0.95	0.17	30.11	<0.001	2.59 (1.84–3.63)
Meary’s Angle (>4.9)	0.74	0.17	18.69	<0.001	2.09 (1.50–2.91)
Calcaneal Bump Height (≤3.9)	0.63	0.18	12.75	<0.001	1.87 (1.33–2.64)
Fowler and Philip Angle (>61.1)	0.43	0.17	6.46	0.011	1.53 (1.10–2.13)
Constant	−4.05	0.26	249.22	<0.001	0.02

**Table 5:** Comparison of foot anatomical measurements according to flatfoot alignment status. [The *p*-value column shows one-way ANOVA test results (* Kruskal–Wallis test). * Meary’s angle which is presented as median (25th–75th percentile) post-hoc multiple comparisons were performed with Bonferroni correction. All measurement units are in millimeters (mm) or degrees (°)].

Measurement	Flat Foot			*p*-Value	Post-Hoc Adj. *p* Value		
	No (n: 934)	Unilateral (n: 74)	Bilateral (n: 158)		No vs. Uni.	No vs. Bil.	Uni. vs. Bil.
Calcaneal Bump Height	4.0 ± 1.2	3.6 ± 1.2	3.5 ± 1.4	<0.001	0.023	<0.001	1.00
Calcaneal Height	45 ± 4	44 ± 3	44 ± 4	<0.001	1.00	<0.001	0.253
Calcaneal Length	94 ± 7	95 ± 7	95 ± 7	0.362	0.812	0.933	1.00
Navicular Height	55 ± 7	51 ± 7	48 ± 7	<0.001	<0.001	<.001	0.003
Tibiocalcaneal Angle	60 ± 9	65 ± 8	67 ± 9	<0.001	<0.001	<0.001	0.112
Bohler’s Angle	35 ± 6	33 ± 5	33 ± 6	<0.001	0.003	0.002	1.00
Fowler and Philip Angle	60 ± 6	62 ± 6	62 ± 6	<0.001	0.014	0.001	1.00
Gissane’s Angle	104 ± 9	105 ± 8	105 ± 9	0.727	1.00	1.00	1.00
Meary’s Angle *	3.1 (1.8–5.1)	3.2 (2.0–6.2)	5.0 (2.1–8.5)	<0.001	0.536	<0.001	0.336

## Data Availability

The data presented in this study are not publicly available due to privacy and ethical restrictions related to patient data.

## Abbreviations

The following abbreviations are used in this manuscript:

PCFD	Progressive Collapsing Foot Deformity
ROC	Receiver Operating Characteristic
AUC	Area Under the Curve
CI	Confidence Interval
IRB	Institutional Review Board
IQR	Interquartile Range
ANOVA	Analysis of Variance
SD	Standard Deviation
VIF	Variance Inflation Factor
CPA	Calcaneal Pitch Angle
HD	Haglund Deformity
